# Rescue of Mutant CFTR Channel Activity by Investigational Co-Potentiator Therapy

**DOI:** 10.3390/biomedicines13010082

**Published:** 2025-01-01

**Authors:** Mafalda Bacalhau, Filipa C. Ferreira, Marcelo Folhadella M. F. Azevedo, Talita P. Rosa, Camilla D. Buarque, Miquéias Lopes-Pacheco

**Affiliations:** 1 Biosystems & Integrative Sciences Institute, Faculty of Sciences, University of Lisbon, 1749-016 Lisbon, Portugal; 2 Department of Chemistry, Pontifical Catholic University of Rio de Janeiro, Rio de Janeiro 22541-041, Brazil

**Keywords:** CFTR modulator, cystic fibrosis, drug development, potentiator, rare mutation, R334W, theratyping, hydroxy-1,2,3-triazole, 1,3-dipolar cycloaddition

## Abstract

**Background:** The potentiator VX-770 (ivacaftor) has been approved as a monotherapy for over 95 cystic fibrosis (CF)-causing variants associated with gating/conductance defects of the CF transmembrane conductance regulator (CFTR) channel. However, despite its therapeutic success, VX-770 only partially restores CFTR activity for many of these variants, indicating they may benefit from the combination of potentiators exhibiting distinct mechanisms of action (i.e., co-potentiators). We previously identified **LSO-24**, a hydroxy-1,2,3-triazole-based compound, as a modest potentiator of p.Arg334Trp-CFTR, a variant with a conductance defect for which no modulator therapy is currently approved. **Objective/Methods:** We synthesized a new set of LSO-24 structure-based compounds, screened their effects on p.Arg334Trp-CFTR activity, and assessed the additivity of hit compounds to VX-770, ABBV-974, ABBV-3067, and apigenin. After validation by electrophysiological assays, the most promising hits were also assessed in cells expressing other variants with defective gating/conductance, namely p.Pro205Ser, p.Ser549Arg, p.Gly551Asp, p.Ser945Leu, and p.Gly1349Asp. **Results:** We found that five compounds were able to increase p.Arg334Trp-CFTR activity with similar efficacy, but slightly greater potency promoted by LSO-150 and LSO-153 (EC_50_: 1.01 and 1.26 μM, respectively). These two compounds also displayed a higher rescue of p.Arg334Trp-CFTR activity in combination with VX-770, ABBV-974, and ABBV-3067, but not with apigenin. When tested in cells expressing other CFTR variants, LSO-24 and its derivative LSO-150 increased CFTR activity for the variants p.Ser549Arg, p.Gly551Asp, and p.Ser945Leu with a further effect in combination with VX-770 or ABBV-3067. No potentiator was able to rescue CFTR activity in p.Pro205Ser-expressing cells, while p.Gly1349Asp-CFTR responded to VX-770 and ABBV-3067 but not to LSO-24 or LSO-150. **Conclusions:** Our data suggest that these new potentiators might share a common mechanism with apigenin, which is conceivably distinct from that of VX-770 and ABBV-3067. The additive rescue of p.Arg334Trp-, p.Ser549Arg-, p.Gly551Asp-, and p.Ser945Leu-CFTR also indicates that these variants could benefit from the development of a co-potentiator therapy.

## 1. Introduction

Cystic fibrosis (CF) is a potentially lethal genetic disease caused by bi-allelic pathogenic variants on the gene encoding the CF transmembrane conductance regulator (CFTR) protein, a cAMP-dependent chloride (Cl^−^) and bicarbonate (HCO_3_^−^) channel expressed at the apical membrane of various epithelial cells in the respiratory, gastrointestinal, and reproductive tracts, among others [1]. The loss of CFTR function causes a broad range of clinical manifestations, with the progression of severe sinopulmonary disease being the primary cause of morbidity and mortality in people with CF (PwCF) [2,3].

CFTR (or ABCC7) is the unique member of the ATP-binding cassette transporter superfamily that functions as an anion channel [4]. It is structurally composed of two homologous halves, each containing a transmembrane domain (TMD) followed by a nucleotide-binding domain (NBD). Each TMD consists of six segments that are assembled to form a pore presenting a selectivity filter that determines CFTR permeability. Anion flow through CFTR is regulated by ATP binding and hydrolysis at the interface between the two NBDs. Both halves of the protein are joined by a disordered regulatory domain whose phosphorylation stimulates channel gating [4]. In order to attain its functional native conformation, CFTR is subjected to a complex stepwise folding and assembly process [5]. However, many of the 2100 CFTR variants cause a defective CFTR-mediated anion transport [6,7], leading to rheological alterations in epithelial secretions [2,3].

The treatment for many PwCF has been significantly transformed over the last decade by the clinical approval of CFTR modulators—i.e., specialized small molecules able to target the fundamental cause of CF [7]. The potentiator VX-770 (or ivacaftor) is a therapeutic agent that directly binds to the CFTR protein at the plasma membrane [8,9] and enhances its activity [10]. It was initially approved as a monotherapy for PwCF harboring at least one copy of p.Gly551Asp-CFTR (legacy G551D)—the prototypic variant with a severe gating defect [11]—and its clinical efficacy was subsequently assessed and confirmed for other variants with a similar defect [12,13]. The long-term clinical data also demonstrated that VX-770 monotherapy achieves sustained benefits, including better-preserved lung function and lower frequencies of pulmonary exacerbations and hospitalizations [14]. Mechanistically, VX-770 promotes CFTR-dependent Cl^−^ transport by increasing the channel open probability [9,10]. Upon PKA-mediated phosphorylation in CFTR, VX-770 increases the frequency and duration of the channel opening in an ATP-independent manner that enables anion transport through the channel pore [15,16]. However, electrophysiological studies indicated that VX-770 treatment only partially restores the p.Gly551Asp-CFTR gating defect [10,16] and a further rescue of CFTR activity could be attained by the combination of potentiators exhibiting distinct mechanisms of action (i.e., co-potentiators) [17,18,19].

Due to the development of translational models and continuous theratyping efforts—i.e., matching the responsive variants with clinically approved modulators—over 95 CFTR variants are now included in the label extension of VX-770 monotherapy [20,21,22,23,24,25]. However, many PwCF harboring (ultra)rare variants with gating/conductance defects remain without an effective modulator therapy. One such variant is p.Arg334Trp (c.1000C>T; legacy R334W), which has a relatively high prevalence in PwCF in Portugal and Brazil (>6% and 2.3% of CF alleles, respectively) [26,27], although it is found at a much lower frequency globally (~0.4% of CF alleles) [6]. This missense variant is located in the sixth segment in TMD1 of the CFTR protein and reduces the channel function without affecting its folding and trafficking to the plasma membrane [28,29]. Studies have also indicated that its defect is most likely due to a significant reduction in conductance rather than open probability [28,29]. As p.Arg334Trp-CFTR still retains residual CFTR-dependent Cl^−^ transport, PwCF harboring this variant are likely to have pancreatic sufficiency and exhibit a milder CF phenotype [6]. Nevertheless, experimental reports have been controversial regarding p.Arg334WTrp-CFTR responsiveness to VX-770. Using different cell models, some studies demonstrated a modest rescue of p.Arg334WTrp-CFTR by VX-770 [18,30,31,32], while others exhibited an unsuccessful increase in CFTR activity [17,21,33].

We previously conducted a screening of a small compound library and identified **LSO-24**, a hydroxyl-1,2,3-triazole derivative, as a promising candidate that was able to enhance p.Arg334Trp-CFTR function with a suboptimal efficacy and potency in both heterologous systems and in intestinal organoids from a p.Arg334Trp-homozygous CF subject [34]. Aiming to improve the potentiator activity, we performed chemical modifications on the LSO-24 structure and screened the novel compounds in CF bronchial epithelial (CFBE41o^−^) cells co-expressing the halide sensitive-yellow fluorescence protein (HS-YFP) and p.Arg334Trp-CFTR. The additivity of the active compounds to other known CFTR potentiators (VX-770, ABBV-974 [formerly GLPG1837], ABBV-3067 [or navocaftor], and apigenin) was assessed and validated by electrophysiological measurements. Finally, we assessed the effects of the active compounds alone and in combination with VX-770 or ABBV-3067 to co-potentiate CFTR activity for other variants with gating/conductance defects, namely p.Pro205Ser, p.Ser549Arg, p.Gly551Asp, p.Ser945Leu, and p.Gly1349Asp (legacy P205S, S549R, G551D, S945L and G1349D).

## 2. Materials and Methods

### 2.1. Chemicals and Compounds

The compounds under study were synthesized by the *Laboratório de Síntese Orgânica* (LabSint) from PUC-Rio and structurally characterized as reported previously [35]. Briefly, the desired acetophenone (**1**, 0.5 mmol), *N*,*N*-dimethylformamide dimethyl acetal (DMA-DMF, 1.0 mmol), and suitable azidobenzene (**2**, 0.75 mmol) were combined in a vial equipped with a magnetic stirring bar and wrapped in aluminum foil. The mixture was heated at 150 °C for 2 h. Thereafter, the reaction mixture was poured into ice-cold ethanol and allowed to precipitate in the refrigerator. The resulting 4-acyl-1,2,3-triazole (**3**) was vacuum filtered, washed with ice-cold ethanol, and dried under reduced pressure.

**Scheme 1:**
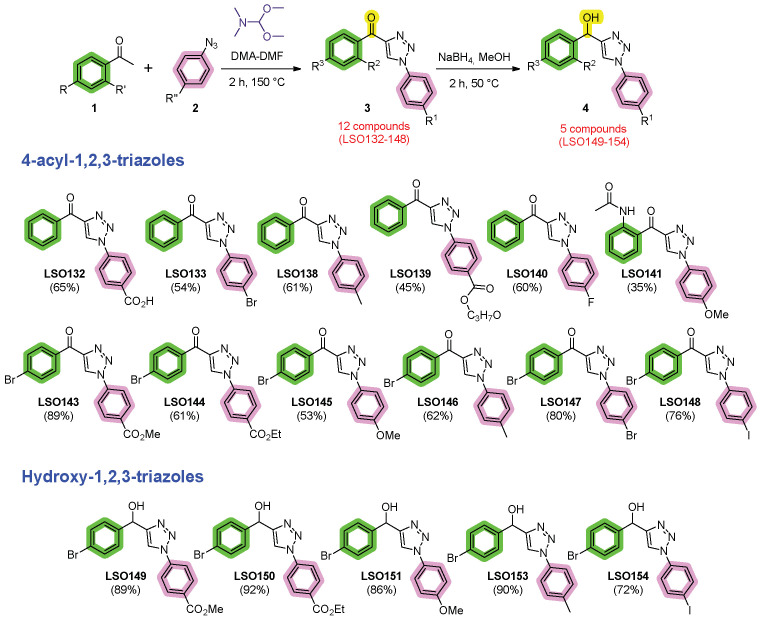
Metal-free synthesis of 4-acyl-1,2,3-triazoles and hydroxy-1,2,3-triazoles for the screening of new CFTR potentiators.

For hydroxy-1,2,3-triazole (**4**) synthesis, 100 mg of selected **3** was added to 5 mL of methanol and stirred for a few minutes in a round-bottom flask. Thereafter, 40 mg of NaBH_4_ was carefully added, and the reaction mixture was refluxed for 2 h. The reaction mixture was then poured into an ice-cold 30% HCl solution. The resulting product was vacuum-filtered, washed with ice-cold water, and dried under reduced pressure. Scheme 1 depicts the chemical structures of compounds used in this study.

Other compounds were purchased from MedChemExpress (Monmouth Junction, NJ, USA) at the highest purity available: apigenin (#HY-N1201), ABBV-974 (#HY-111099), ABBV-3067 (#HY-109152), VX-770 (#HY-13017), forskolin (Fsk; #HY-15371), and CFTR inhibitor-172 (Inh172, #HY-16671). All compounds were diluted in dimethyl sulfoxide (DMSO) and stored at −20 °C.

### 2.2. Cell Culture

Parental CFBE41o^−^ cells stably expressing HS-YFP (p.His148Gln-Ile152Leu) were cultured in minimum essential medium (MEM, #10-010-CV, Corning, VA, USA) supplemented with 10% fetal bovine serum (#LTI 10270-106, Gibco, Carlsbad, CA, USA) [36]. CFBE41o^−^ cells stably co-expressing HS-YFP (p.Phe46Leu-His148Gln-Ile152Leu) and p.Arg334Trp-CFTR were cultured in a similar medium including 2 µg mL^−1^ puromycin (#P8833, Sigma-Aldrich, St. Louis, MO, USA) [34]. For the YFP assay of CFTR activity, cells were plated (30,000 cells/well) on 96-well clear-bottom, black-walled microplates (#655090, Greiner Bio-One, Kremsmünster, Austria). All cells were maintained in a humidified incubator (5% CO_2_) at 37 °C.

### 2.3. Site-Directed Mutagenesis and Transient Transfection

Site-directed mutagenesis was performed using KOD HOT start DNA polymerase (#71086-3, Sigma-Aldrich, St. Louis, MO, USA), according to manufacturer’s instructions, to insert variants under study (c.613C>T or p.Pro205Ser; c.1645A>C or p.Ser549Arg; c.1647T>G or p.Ser549Arg; c.1652G>A or p.Gly551Asp; c.2834C>T or p.Ser945Leu; and c.4046G>A or p.Gly1349Asp) into CFTR cDNA cloned in the pcDNA5 expression vector. After confirmation by Sanger sequencing, CFBE41o^−^ cells expressing HS-YFP seeded onto 96-well plates were transfected with 0.1 µg per well of the indicated plasmid. Lipofectamine 3000 (#L3000015, ThermoFischer Scientific, Waltham, MA, USA) was used as the transfection agent diluted in Opti-MEM reduced serum medium (#31985070, ThermoFischer Scientific, Waltham, MA, USA) according to manufacturer’s instructions.

### 2.4. HS-YFP Assay on a Plate Reader

CFTR activity was determined by the HS-YFP microfluorimetric assay (described in detail in previous studies [34,36]) using CFBE41o^−^ cells expressing HS-YFP together with stably expressed p.Arg334Trp-CFTR or transiently transfected with CFTR cDNA (variants p.Pro205Ser, p.Ser549Arg, p.Gly551Asp, p.Ser945Leu, and p.Gly1349Asp) cloned into pcDNA5. Briefly, cells were washed with phosphate-buffered saline (PBS; in mM: 137 NaCl, 2.7 KCl, 8.1 Na_2_HPO_4_, 1.5 KH_2_PO_4_, 1 CaCl_2_, and 0.5 MgCl_2_) and then incubated for 30 min at 37 °C with 60 μL of PBS plus Fsk (5 μM) and test compound(s) to stimulate CFTR channels. DMSO (vehicle) and VX-770 (1 μM) were used as negative and positive controls, respectively. All conditions were performed in triplicate on each plate. Thereafter, cells were transferred to a microplate reader (Tecan Infinite 200 Pro) equipped with high-quality excitation (485 ± 20 nm) and emission (535 ± 25 nm) filters for YFP. Each reading consisted of a continuous 14 s fluorescence recording with 2 s before and 12 s after injection of 165 μL of an iodide-containing solution (PBS with Cl^−^ replaced by I^−^; final I^−^ concentration: 100 mM). Data were normalized to the initial background-subtracted fluorescence. To determine the I^−^ influx rate, the final 11 s of the data for each well were fitted with an exponential function to extrapolate the initial slope (dF/dt).

### 2.5. In Silico Absorption, Distribution, Metabolism, and Excretion (ADME) Analysis

The online software SwissADME was used to perform the in silico ADME analysis as described previously [37]. Based on Lipinski’s rule of five [38], the following physicochemical parameters were assessed: molecular weight (MW), number of H-bond donors (nHBD), number of H-bond acceptors (nHBA), number of rotatable bonds (nRB), and the calculated logarithm of the octanol-water partition coefficient (LogP). The topological polar surface area (TPSA) was also assessed. Finally, the assessment of potential “promiscuous” substructures that demonstrate responses in bioassays independent of the target was assessed by using filters for the identification of pan-assay interference compounds (PAINS), as noted before [39].

### 2.6. Micro-Ussing Chamber Measurements

CFBE41o^−^ cells expressing p.Arg334Trp-CFTR were seeded onto collagen IV-coated Snap-well filter inserts (#734-1646, Corning, New York, NJ, USA) to establish polarized monolayers. When the transepithelial electrical resistance of cells was ≥1000 Ω·cm^2^, micro-Ussing chambers were mounted to measure transepithelial voltage (V_te_) and transepithelial resistance (R_te_), as noted before [40,41]. Briefly, after the equilibration period, baseline values were recorded and compounds were added sequentially: Fsk (0.128 μM), test compound(s), and Inh-172 (30 μM). Recorded V_te_ and R_te_ were used to calculate the equivalent short-circuit current (Isc_eq_) using Ohm’s law: Isc = V_te_/R_te_ [42].

### 2.7. Statistical Analysis

All conditions were performed in at least three independent experiments. Data are depicted as mean ± standard deviation (SD) in scatter plots with bars. All statistical analyses were performed using GraphPad Prism software version 8.3 (GraphPad Inc., San Diego, CA, USA). Statistical comparisons between the conditions tested were assessed using one-way ANOVA followed by Dunnett’s or Tukey’s posthoc tests, and values of *p* < 0.05 were considered significant.

## 3. Results

### 3.1. Screening of New Compounds as p.Arg334Trp-CFTR Potentiators

**Figure 1:**
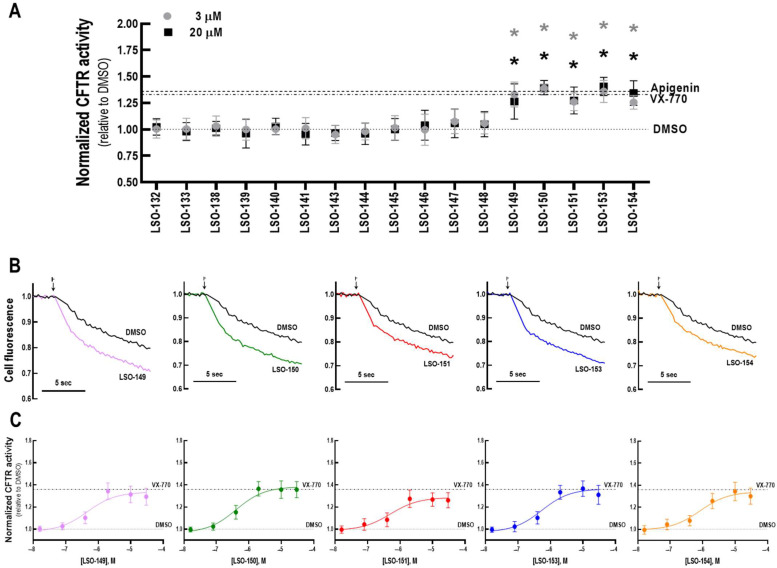
**Search for new p.Arg334Trp-CFTR potentiators using the HS-YFP assay on a plate reader**. CFBE41o^−^ cells co-expressing HS-YFP and p.Arg334Trp-CFTR were acutely incubated (30 min) with Fsk (5 μM) plus test compound. DMSO (vehicle) was used as a negative control, while VX-770 (1 μM) and apigenin (50 μM) were used as positive controls. (**A**) CFTR activity was assessed based on the cell-fluorescence quenching rate and normalized to the negative control: * *p* < 0.05 vs. DMSO. (**B**) Representative cell fluorescence recording on a plate reader. The arrow depicts the time of injection of an I^−^-containing solution. (**C**) Dose-response relationships for active compounds: LSO-149, LSO-150, LSO-151, LSO-153, and LSO-154. Data are depicted as means ± SD of 4 independent experiments.

All the compounds (Scheme 1) were tested in CFBE41o^−^ cells co-expressing HS-YFP and p.Arg334Trp-CFTR to assess CFTR-mediated I^−^ influx based on fluorescence quenching. The cells were incubated for 30 min with Fsk (an adenyl cyclase activator that increases intracellular cAMP levels) plus the test compounds in two different concentrations (3 and 20 μM). Thereafter, the efficacy of the compounds in rescuing CFTR activity was determined by the YFP quenching rate and normalized to DMSO (negative control). Among 17 compounds, five were demonstrated to enhance p.Arg334Trp-CFTR activity (Figure 1A,B). At both concentrations, the treatment with LSO-149, LSO-150, LSO-151, LSO-153, and LSO-154 caused an increase in CFTR-mediated I^−^ influx compared to that of the cells treated with DMSO. Such an increase was equivalent to that of the cells treated with VX-770 or apigenin (used as positive controls).

Next, the active compounds were submitted for further analysis for evaluation of their potency and efficacy. To this aim, the compounds were retested as potentiators at various concentrations in the range 0.16–30 μM with Fsk on CFBE41o^−^ cells. The compounds were found to be active in the low micromolar range with slightly different potency (EC_50_ = 1.46 μM LSO-149, 1.01 μM LSO-150, 1.84 μM LSO-151, 1.26 μM LSO-153, and 2.07 μM LSO-154) (Figure 1C).

### 3.2. In Silico ADME Analysis

As an important step in early drug development phases for novel molecules designed to eventually become therapies, we determined the theoretical ADME properties of the active compounds by in silico analysis. Based on Lipinski’s rule of five [38], an orally active small molecule should have MW ≤ 500 Da, nHBD ≤ 5, nHBA ≤ 5, nHRB ≤ 10, and LogP ≤ 5. Absorption and membrane permeability are also influenced by TPSA, which should be ≤140 Å^2^ to have intestinal absorption. The results are depicted in Table 1, together with the values obtained for VX-770 as the reference potentiator. All five active compounds follow Lipinski’s rule of five and have a TPSA < 140 Å^2^, indicating they have good gastrointestinal absorption. Furthermore, the compounds LSO-149, LSO-150, LSO-151, LSO-153, and LSO-154 were not recognized by the SwissADME software as PAINS, which enables us to rule out a non-specific interaction or a false positive biological output.

### 3.3. Compound Assessment for p.Arg334Trp-CFTR Co-Potentiator Activity

**Figure 2:**
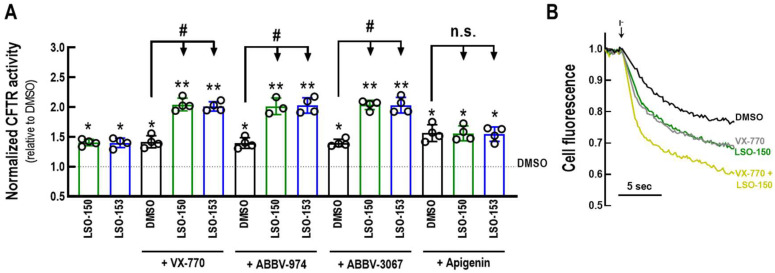
**Functional evaluation of single and combined potentiator treatment on CFTR-mediated I^−^ influx**. (**A**) CFBE41o^−^ cells co-expressing HS-YFP and p.Arg334Trp-CFTR were acutely incubated (30 min) with Fsk (5 μM) plus test compounds (individually or in combination): LSO-150 (3 μM); LSO-153 (5 μM); VX-770 (1 μM); ABBV-974 (10 μM); ABBV-3067 (10 μM); and apigenin (50 μM). DMSO (vehicle) was used as the negative control. CFTR activity was assessed based on the cell-fluorescence quenching rate and normalized to the negative control. Data are depicted as means ± SD of 4 independent experiments: * *p* < 0.05; ** *p* < 0.01 vs. DMSO; ^#^ *p* < 0.05 vs. single potentiator; n.s.—not significant. (**B**) Representative cell fluorescence recording on a plate reader. The arrow depicts the time of injection of an I^−^-containing solution.

As LSO-150 and LSO-153 demonstrated a slightly greater potency compared to the other active compounds (i.e., LSO-149, LSO-151, and LSO-154), we used those two for subsequent experiments. We then interrogated whether a further enhancement of CFTR activity could be attained by the combined treatment of LSO-150 or LSO-153 with known CFTR potentiators, namely VX-770, ABBV-974, ABBV-3067, and apigenin, on CFBE41o^−^ cells co-expressing HS-YFP and p.Arg334Trp-CFTR. Upon Fsk stimulation, a further increase in the fluorescence quenching rate was observed in the cells treated with LSO-150 or LSO-153 in combination with VX-770, ABBV-974, or ABBV-3067 (Figure 2). However, the combination of LSO-150 or LSO-153 with apigenin exhibited an equivalent cell-fluorescence quenching rate to that of each compound individually.

**Figure 3:**
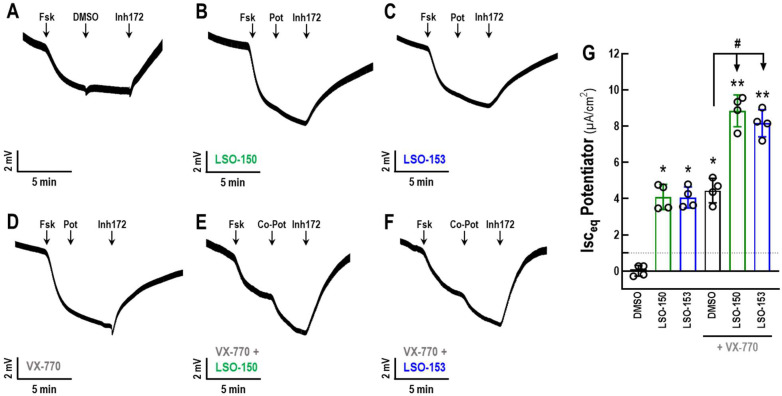
**Functional evaluation of single and combined potentiator treatment on CFTR-mediated current by micro-Ussing chamber measurements**. (**A**–**F**) Representative tracings of polarized monolayers of CFBE41o^−^ cells expressing p.Arg334Trp-CFTR in micro-Ussing chambers. During the recordings, cells were sequentially treated (as depicted by arrows) with Fsk (0.128 μM), tested compounds ([**A**] DMSO; [**B**] 3 μM LSO-150; [**C**] 5 μM LSO-153; [**D**] 3 μM VX-770; [**E**] 3 μM VX-770 + 3 μM LSO-150; or [**F**] 3 μM VX-770 + 5 μM LSO-153) and Inh-172 (30 μM). (**G**) Data are depicted as mean ± SD of 4 independent experiments. Data reported are the amplitude of the Isc_eq_ elicited by single or combined potentiators after CFTR stimulation with Fsk: * *p* < 0.05; ** *p* < 0.01 vs. DMSO; ^#^ *p* < 0.05 vs. single potentiator.

Next, we validated the data obtained by the HS-YFP assay by measuring short-circuit currents in the polarized monolayers of CFBE41o^−^ cells expressing p.Arg334Trp-CFTR to assess the alterations in the CFTR-mediated Cl^−^ current. Upon the activation of the cAMP-dependent CFTR-mediated Cl^−^ transport by Fsk, there was a small lumen-negative response, indicating the residual function of p.Arg334Trp-CFTR (Figure 3A–F). Compared to DMSO, the acute treatment with LSO-150, LSO-153, or VX-770 elicited an increase in Isc_eq_ with a further response by the co-administration of VX-770 plus LSO-150 or LSO-153 (Figure 3G). These responses were inhibited by adding Inh172, suggesting they were specific to CFTR currents.

### 3.4. Assessment of Co-Potentiator Activity for Other CFTR Variants with Gating/Conductance Defects

**Figure 4:**
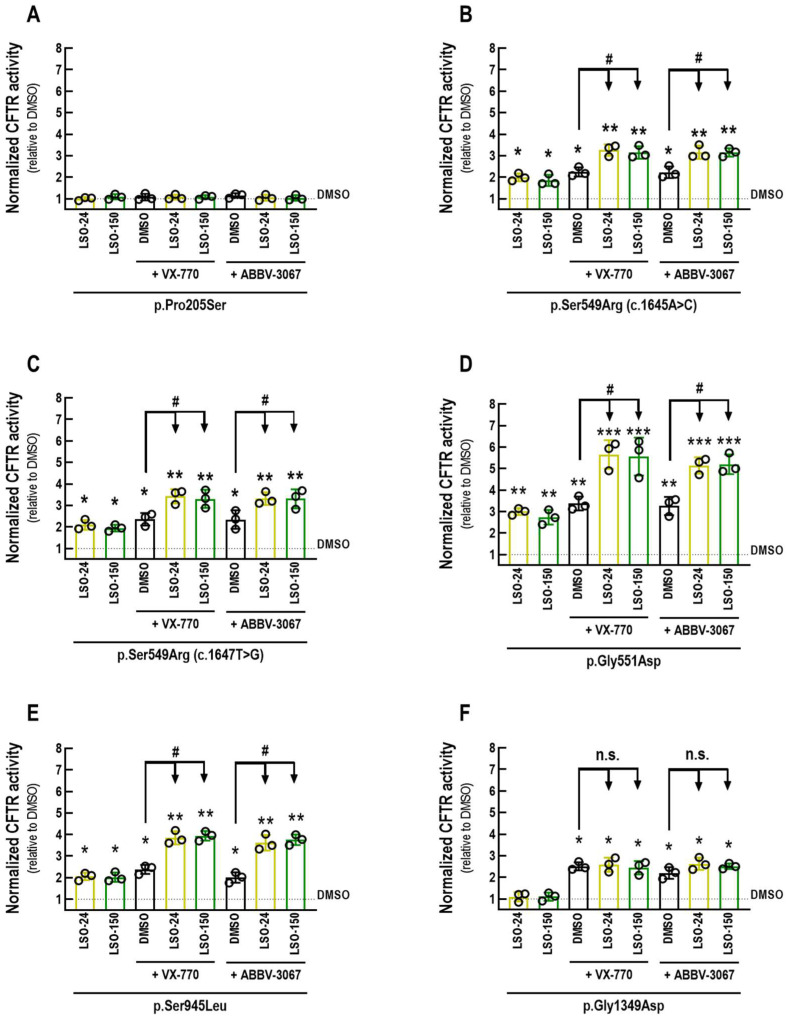
**Functional evaluation of single and combined potentiator treatment on CFTR-mediated I^−^ influx**. CFBE41o^−^ cells stably expressing HS-YFP were transiently transfected with CFTR cDNA containing variants (**A**) p.Pro205Ser, (**B**) p.Ser549Arg [c.1645A>C], (**C**) p.Ser549Arg [c.1647T>G], (**D**) p.Gly551Asp, (**E**) p.Ser945Leu, or (**F**) p.Gly1349Asp. After 24 h, cells were acutely incubated (30 min) with Fsk (5 μM) plus test compounds (individually or in combination): LSO-24 (5 μM); LSO-150 (3 μM); VX-770 (1 μM); and ABBV-3067 (10 μM). DMSO (vehicle) was used as the negative control. CFTR activity was assessed based on the cell-fluorescence quenching rate and normalized to the negative control. Data are depicted as means ± SD of 3 independent experiments: * *p* < 0.05, ** *p* < 0.01, *** *p* < 0.001 vs. DMSO; ^#^ *p* < 0.05 vs. single potentiator; n.s.—not significant.

As CFTR variants with similar defect(s) may respond differently to the same treatment(s), we assessed whether LSO-24 or its derivative LSO-150, alone or in combination(s), could rescue CFTR activity for other variants with gating/conductance defects. To this aim, CFBE41o^−^ cells stably expressing HS-YFP were transiently transfected with CFTR cDNA containing the variants under study (p.Pro205Ser, p.Ser549Arg, p.Gly551Asp, p.Ser945Leu, or p.Gly1349Asp). After 24 h, the cells were acutely stimulated with Fsk plus test compound(s), and the cell fluorescence was recorded before and after the addition of an I^−^-containing solution. The net effect elicited by LSO-24 or LSO-150 was increased by the co-administration of VX-770 or ABBV-3067 in cells expressing p.Ser549Arg (c.1645A>C or c.1647T>G), p.Gly551Asp, and p.Ser945Leu (Figure 4B–E), suggesting co-potentiator activity for these CFTR variants. In p.Gly1349Asp-expressing cells, VX-770 or ABBV-3067 increased the fluorescence quenching rate with no effect promoted by LSO-24 or LSO-150, alone or in combination(s) (Figure 4F). However, none of these compounds was able to rescue CFTR activity in p.Pro205Ser-expressing cells (Figure 4A).

## 4. Discussion

Even though six CFTR modulator compounds are now approved for clinical use (two potentiators and four correctors), these are unable to fully restore mutant CFTR folding and activity, even when used in combinations [10,43,44,45]. Accordingly, the identification of novel small molecules that are able to rescue the activity of the mutant CFTR remains of high relevance for designing improved pharmacological strategies to treat the fundamental cause of CF [46,47]. In this study, we started from the chemical structure of LSO-24 [34] to synthesize a small collection of new triazole derivatives. The 4-acyl-1,2,3-triazoles (**3**) are key intermediates for synthesizing hydroxy-1,2,3-triazoles (**4**). These compounds were synthesized using a solvent- and metal-free telescopic method, employing commercial acetophenones (**1**) and arylazides (**2**) in the presence of DMA-DMF, as previously reported [35]. Notably, bromine substitution patterns were chosen due to their established biological significance, particularly concerning CFTR variants [34,35,40]. A total of 12 compounds were initially produced with yields ranging from 35% to 89%. The selected compounds were subsequently subjected to acyl reduction using NaBH_4_, resulting in five hydroxy-1,2,3-triazoles (**4**) with yields between 72% and 92%, as summarized in Scheme 1. After compound synthesis and screening, we found that the hydroxy-1,2,3-triazoles (LSO-149, LSO-150, LSO-151, LSO-153, and LSO-154) were able to rescue p.Arg334Trp-CFTR activity in cell lines with additive effects when combined with VX-770, ABBV-974, or ABBV-3067. LSO-24 and its analog LSO-150 also rescued other CFTR variants with gating/conductance defects (p.Ser549Arg, p.Gly551Asp, and p.Ser945Leu) and were demonstrated to follow Lipinski’s rule of five for drug-like molecules [38], indicating that they have good bioavailability.

Various CFTR potentiators have been identified by either high-throughput screening campaigns [10,48,49,50] or hypothesis-driven studies [51,52,53]. However, only a few CFTR potentiators attained clinical testing and VX-770 was the only one approved for PwCF with specific genotypes until recently. VX-445 (or elexacaftor) was clinically approved as a corrector in a triple combinatorial therapy with VX-661 and VX-770 [54,55,56]; however, recent evidence demonstrated that VX-445 exhibits a dual corrector and weak potentiator activity for several CFTR variants [57,58]. Nevertheless, it was previously found to be inefficient in potentiating p.Arg334Trp-CFTR function in cell lines [34] and demonstrated no additive effects to VX-770 in intestinal organoids harboring p.Arg334Trp on one allele and a minimal function variant in trans [31,32]. Among other CFTR potentiators tested in clinical studies, ABBV-974 [59] and VX-561 (or deutivacaftor; formerly CTP-656) [60] share a common mechanism with VX-770 [48,61]. Indeed, VX-561 is a deuterated form of VX-770 with an improved stability and half-life [61] that was recently approved for clinical use in a new triple combinatorial therapy with VX-661 and VX-121 (or vanzacaftor) [60], while VX-770 and ABBV-974 were found to compete for a binding site in a cleft generated by the transmembrane segments 4, 5, and 8 [8]. Their binding at this site facilitates the channel gating in an ATP-independent manner [15,62]. ABBV-3067 is also expected to act like VX-770, since ABBV-3067 is derived from the same chemical series of GLPG2451 and these compounds exert similar biological activity [48,50]. On the other hand, despite their suboptimal efficacy, our compounds (LSO-150 and LSO-153) appear to potentiate p.Arg334Trp-CFTR activity by a distinct mechanism from that of VX-770. Likewise, genistein and its analog apigenin potentiate CFTR channel gating by an alternative mechanism to that of VX-770 [49,52,53,63]. Early in silico studies suggested that these compounds can potentiate CFTR channel gating by binding at the NBD1:NBD2 dimer interface, thus accelerating the channel opening and delaying its closure [63].

Combinatorial profiling and clustering analysis have suggested the existence of at least three mechanistically different groups of CFTR potentiators, likely having distinct binding sites [17,47,58]. As they can be targeted simultaneously to maximize the channel gating of mutant CFTR, co-potentiation has emerged as a therapeutic strategy for CFTR variants that are poorly responsive to single potentiators [17,49]. Regarding p.Arg334WTrp-CFTR, there are controversial reports about its responsiveness to VX-770 monotherapy. In Fischer rat thyroid (FRT) cells, VX-770 failed to increase p.Arg334Trp-CFTR activity [17,21], whereas a small increase in CFTR activity was found in p.Arg334Trp-harboring CFBE41o^−^ cells [18] and in intestinal organoids [30,31], and improved lung function and nutritional status was described in a case report (p.Arg334Trp/p.Tyr515Ter genotype) [32]. Likewise, we observed a small rescue of p.Arg334Trp-CFTR activity in CFBE41o^−^ cells by VX-770 that was further increased by the co-potentiator LSO-24 and subsequently confirmed in the intestinal organoids of a p.Arg334Trp-homozygous CF subject [34]. In the current study, the investigational compounds LSO-150 and LSO-153 were able to increase p.Arg334Trp-CFTR activity with a further rescue in combination with VX-770, ABBV-974, or ABBV-3067. A response to apigenin was also detected in p.Arg334Trp-CFTR activity but with no additive effect in combination with LSO-150 or LSO-153, suggesting they might share a common mechanism for CFTR potentiation.

CFTR variants may respond differently to the same potentiator, so we investigated whether LSO-24 and its analog LSO-150 could rescue other variants with gating/conductance defects, namely p.Pro205Ser, p.Ser549Arg, p.Gly551Asp, p.Ser945Leu, and p.Gly1349Asp. VX-770 monotherapy is approved for all these variants, except p.Pro205Ser [23]. This variant is present in the third transmembrane segment of TMD1 and is associated with a milder clinical phenotype and pancreatic sufficiency, suggesting that it might retain residual CFTR-dependent Cl^−^ transport [64]. However, early studies indicated that p.Pro205Ser-CFTR exhibits a predominant protein processing defect [65]. A recent case series also demonstrated that the triple combination VX-445/VX-661/VX-770 promotes therapeutic benefits for PwCF harboring p.Pro205Ser on one allele and a minimal function variant in trans [66]. The findings suggest that correctors are needed to rescue this variant, thus explaining the lack of effect of our compounds on CFTR activity either as single agents or as co-potentiators. On the other hand, the p.Ser945 residue, located in the transmembrane segment 8 in TMD2, is suggested to contribute to ion selectivity [67] and be in a hotspot on CFTR for potentiation [8]. PwCF harboring p.Ser549Arg-CFTR are reported to have a milder disease with pancreatic sufficiency [6,68]. In our experiments, this variant was demonstrated to be responsive to either LSO-24 or LSO-150 with a greater rescue of CFTR activity in combination with VX-770 or ABBV-3067.

Both p.Ser549Arg and p.Gly551Asp are CFTR variants present in NBD1. Interestingly, the former can be caused by two distinct nucleotide substitutions (c.1645A > C and c.1647T > G) [6]. We, therefore, assessed whether differences in the tRNA abundance related to this missense variant could play a role in its pharmacological modulation, as it was observed for other variants [69]; however, equivalent results were found for the potentiators tested. Indeed, our data demonstrated the rescue of CFTR activity for p.Ser549Arg and p.Gly551Asp by LSO-24 or LSO-150 with a further increase in combination with VX-770 or ABBV-3067. A partial restoration of p.Ser549Arg-, p.Gly551Asp-, and p.Ser945Leu-CFTR activity was also reported in previous studies of single potentiators [18,21,49,68], suggesting that PwCF harboring these variants are likely to benefit from co-potentiator therapy.

p.Gly1349Asp is located in NBD2 in an equivalent position to p.Gly551Asp in NBD1. While both of the variants were rescued by VX-770 and ABBV-3067, the compounds LSO-24 and LSO-150 failed to increase p.Gly1349Asp-CFTR activity. Likewise, previous studies demonstrated that genistein was inefficient in rescuing CFTR activity for this variant [70]. Despite the symmetrical positions of p.Gly551Asp and p.Gly1349Asp, these variants are also reported to exhibit distinct gating defects and pharmacological profiles [71,72], indicating that different co-potentiators may be needed to maximize their CFTR activity.

## 5. Conclusions

We found that the hydroxy-1,2,3-triazole may represent a suitable chemical series for the design of novel CFTR potentiators with the ability to rescue not only p.Arg334Trp but also other variants with a gating/conductance defect (p.Ser549Arg, p.Gly551Asp, and p.Ser945Leu). While additional mechanistic experiments should be performed in subsequent studies, the lack of additive potentiation of CFTR activity by LSO-24 or its analog LSO-150 to apigenin suggests that they act by a similar mechanism, which is conceivably distinct from that of VX-770 and ABBV-3067. Since these new co-potentiators were identified within a relatively small set of analogs, further exploitation of this chemical series may lead to compounds with improved efficacy and potency to rescue mutant CFTR activity.

## Figures and Tables

**Table 1:** Physicochemical variables and in silico ADME analysis of active compounds.

Compound	MW	nHBD	nHBA	nRB	LogP	TPSA	GI Absorption	PAINS Alert
LSO-149	388.22	1	5	5	2.76	77.24	High	0
LSO-150	402.24	1	5	6	3.06	77.24	High	0
LSO-151	360.21	1	4	4	2.80	60.17	High	0
LSO-153	344.21	1	3	3	3.15	50.94	High	0
LSO-154	456.08	1	3	3	3.50	50.94	High	0
VX-770	392.49	3	3	5	4.47	82.19	High	0

Abbreviations: MW: molecular weight; nHBD: number of H-bond donors; nHBA: number of H-bond acceptors; nRB: number of rotatable bonds; LogP: values correspond to Consensus LogPw/o; TPSA: topological polar surface area; GI: gastrointestinal; PAINS: pan-assay interference structures.

## Data Availability

The original contributions presented in the study are included in the article, further inquiries can be directed to the corresponding author.

## Abbreviations

ADME—absorption, distribution, metabolism, and excretion; ATP—adenosine triphosphate; cAMP—cyclic adenosine monophosphate; CF—cystic fibrosis; CFBE41o^−^—CF bronchial epithelia; CFTR—CF transmembrane conductance regulator; DMSO—dimethyl sulfoxide; Fsk—forskolin; FRT—Fischer rat thyroid; HS-YFP—halide sensitive yellow fluorescence protein; Isc_eq_—equivalent short-circuit current; LogP—values correspond to Consensus LogPw/o; MEM—minimum essential medium; MW—molecular weight; NBD—nucleotide-binding domain; nHBA—number of H-bond acceptors; nHBD—number of H-bond donors; nRB—number of rotatable bonds; PAINS—pan-assay interference structures; PBS—phosphate-buffered saline; PKA—protein kinase A; PwCF—people with CF; R_te_—transepithelial resistance; SD—standard deviation; TMD—transmembrane domain; TPSA—topological polar surface area; V_te_—transepithelial voltage.
